# Genome-Wide Comprehensive Analysis the Molecular Phylogenetic Evaluation and Tissue-Specific Expression of SABATH Gene Family in *Salvia miltiorrhiza*

**DOI:** 10.3390/genes8120365

**Published:** 2017-12-05

**Authors:** Bin Wang, Shiqiang Wang, Zhezhi Wang

**Affiliations:** 1 National Engineering Laboratory for Resource Development of Endangered Crude Drugs in Northwest China, Key Laboratory of the Ministry of Education for Medicinal Resources and Natural Pharmaceutical Chemistry, College of Life Sciences, Shaanxi Normal University, Xi’an 710119, China; happywangbin2003@163.com (B.W.); wsq@snnu.edu.cn (S.W.); 2 College of Chemistry, Biology and Materials Science, East China University of Technology, Nanchang 330013, China

**Keywords:** SABATH gene family, phylogenetic analysis, conserved motifs, nonsynonymous and synonymous substitution rate, positive selection, functional divergence, tissue-specific expression

## Abstract

The plant SABATH gene family is a group of *O*-methyltransferases (*O*-MTs), which belongs to the *S*-adenosyl-l-methionine-dependent methyltransferases (SAM-MTs). The resulting reaction products of SABATH genes play an important role in various processes of plant development. In this study, a total of 30 SABATH genes were detected in *Salvia miltiorrhiza*, which is an important medicinal plant, widely used to treat cardiovascular disease. Multiple sequence alignment and phylogenetic analyses showed that *SmSABATH* genes could be classified into three groups. The ratios of non-synonymous (Ka) and synonymous (Ks) substitution rates of 11 pairs paralogous of *SmSABATH* genes revealed that the *SmSABATH* genes had gone through purifying selection. Positive selection analyses using site models and branch-site models indicated that *SmSABATH* genes had undergone selective pressure for adaptive evolution. Functional divergence analyses suggested that the *SmSABATH* subgroup genes were divergent in terms of functions and positive selection sites that contributed to a functional divergence among the subgroups that were detected. Tissue-specific expression showed that the SABATH gene family in *S. miltiorrhiza* was primarily expressed in stems and leaves.

## 1. Introduction

Methylation is a ubiquitous reaction that takes place in bacteria, fungi, plants, and mammals. The process of methylation is catalyzed by *S*-adenosyl-l-methionine-dependent methyltransferases (SAM-MTs), and involves the transfer of the methyl group of *S*-adenosyl-l-methionine (SAM) to carbon, nitrogen, oxygen, or sulfur atoms, and modifies DNA, RNA, proteins, or small molecules with the formation of corresponding methylated products and *S*-adenosyl-l-homocysteine (SAH) [1]. Enzymatic methylation of hydroxyl and carboxyl moieties are catalyzed by *O*-methyltransferases (*O*-MTs) [2], of which there are three defined types, via protein X-ray crystallography [3,4,5]. Type 1 *O*-MTs exclusively methylate oxygen atoms of the hydroxyl moieties of phenylpropanoid-based compounds [6] and type 2 *O*-MTs are specific to phenylpropanoid esters of the coenzyme A, and are found in all lignin-producing plants [3]. The type 3 *O*-MTs specifically methylate carboxyl groups of small molecules, and also the nitrogen atoms of some alkaloids, such as theobromine and caffeine, and they are collectively named “SABATH”, based on the three earliest-identified genes that were belonging to this family, SAMT (salicylic acid carboxyl methyltransferase) [7], BAMT (benzoic acid carboxyl methyltransferase) [8], and theobromine synthase [9].

The SABATH family of methyltransferases are mainly one class of small molecule methyltransferase that are found in plants. The majority of characterized SABATH methyltransferases catalyze the methylation of carboxylic acids [10]. Except for the three first-identified SABATH methyltransferases, more and more other members of this gene family have been found in different species. The jasmonic acid carboxyl methyltransferase (JMT) [11], indole-3-acetic acid carboxyl methyltransferase (IAMT) [12], farnesoic acid carboxyl methyltransferase (FAMT) [13], and gibberellic acid carboxyl methyltransferase (GAMT) [14], were first identified in *Arabidopsis*. Then, cinnamate/*p*-coumarate carboxyl methyltransferase (CCMT) were found in sweet basil (*Ocimum basilicum*) [15]. The loganic acid methyltransferase (LAMT) [16] and anthranilic acid methyltransferase (AAMT) [17] were discovered in *Catharanthus roseus* and maize (*Zea mays*), respectively. In addition to carboxyl methyltransferases, the SABATH family also includes a number of nitrogen methyltransferases that are involved in caffeine biosynthesis, which are homologous to carboxyl SABATH methyltransferases [18]. The primary enzymes contain 7-methylxanthine methyltransferase (MXMT), 3,7-methylxanthine methyltransferase (DXMT), and xanthosine methyltransferase (XMT), which were isolated from tea or coffee plants [19]. Furthermore, researchers also found that a SABATH methyltransferase of *PpSABATH1* from the moss *Physcomitrella patens* could catalyze *S*-methylation of thiols [10].

The substrates of those enzymes are important plant hormones and signaling molecules, such as jasmonic acid (JA), salicylic acid (SA), gibberellic acid (GA), and indole-3-acetic acid (IAA), and these play critical roles in diverse biological processes ranging from plant growth and development to plant interactions with the environment [19]. Using a rapid biochemical assay system for the initial screening of compounds for individual SABATH proteins, there were 59 potential substrates that were known to exist in plants [13]. Furthermore, the resulting reaction products of the SABATH were methyl esters, such as methyl jasmonate, methyl salicylate and methyl benzoate, which often contribute to a plants characteristic scent or flavor and render them appealing to humans, animals, or insects. These products were also found to be involved in the regulation of diverse developmental processes, such as root growth, seed germination, flower or fruit development, and leaf abscission [20,21]. Plants can utilize a variety of mechanisms with those methyltransferases to regulate the concentration of those hormones, signaling molecules, and methyl esters, through biosynthesis, conjugation/deconjugation and degradation to modulate the growth and development of plants [22,23]. For example, IAA-methyltransferase (IAMT1) is involved in plant leaf development by means of regulating IAA homeostasis [12].

With regards to SABATH methyltransferase itself, many characterized SABATH methyltransferases play important biological roles. SABATH methyltransferase mainly functions in plant defense. The first of SAMT gene shown to have a defensive role was *Arabidopsis BSMT1*, which has shown induction as a response to abiotic stress, which participates in direct defense [24]. Then, *OsBISAMT1* of *Oryza sativa* was found to be involved in disease resistance responses, as well as in wound response in rice [25]. Besides, the nitrogen-containing compounds that are produced by SABATHs are toxic to herbivorous insects; therefore, many nitrogen methyltransferases may also have roles in plant defense [26]. Moreover, due to the fact that methyl-*p*-coumarate has been shown to have insecticidal or insect-deterrent as well as antifungal properties [27], CCMTs that are responsible for the formation of methyl-*p*-coumarate in sweet basil (*O. basilicum*) [15] may be related to plant resistance. Analogously, for methyl anthranilate, which might be involved in indirect plant defense, the AAMTs that were identified from maize (*Z. mays*) [17] may also be connected to plant defense responses. In addition, other biological functions of SABATH methyltransferases have been successively discovered. For instance, *Arabidopsis* JMT is a key enzyme for jasmonate-regulated plant responses, and has a function in plant defense against fungi [11]. *Arabidopsis* GAMTs were shown to regulate seed germination [14]. *PgIAMT1*, identified from white spruce (*Picea glauca*), may play a role in embryogenesis, probably via the modulation of the homeostasis of IAA [19]. *PpSABATH1*, isolated from the moss *P. patens*, has a role in detoxification and also in the tolerance to toxic thiols [10].

*Salvia miltiorrhiza* Bunge (“danshen” or “tanshen” in Chinese) is an important medicinal plant and its dry roots or rhizomes have been widely used in Chinese medicines for treating coronary heart disease, hepatitis, menstrual disorders, menostasis, blood circulation diseases, and other cardiovascular diseases [28]. The main bioactive components of *S. miltiorrhiza* include two major groups of active ingredients; one is the water-soluble (hydrophilic) phenolics, such as rosmarinic acid, salvianolic acid A, salvianolic acid B, and lithospermic acid, and the other group is the lipid-soluble (nonpolar, lipophilic) diterpenoids, known as tanshinones [29]. *S. miltiorrhiza* is being developed to serve as a potential medicinal model plant for research on traditional Chinese medicines, because of its remarkable and reliable therapeutic actions [30]. Recently, with the development of high-throughput technologies, the *S. miltiorrhiza* genome was sequenced and assembled. Information regarding the *S. miltiorrhiza* genomic database has been published [31].

Since the genetic background of the *S. miltiorrhiza* genome has become more defined, we can detect more bioinformatics about SABATH gene family in *S. miltiorrhiza*, such as basic biological information, phylogenetic relationships, functional divergence, and so on. Here, using bioinformatics tools and biotechnological means, we identified the SABATH gene family in *S. miltiorrhiza* using the current *S. miltiorrhiza* genome. A phylogenetic tree was constructed to evaluate the evolutionary relationships of *SmSABATH* with SABATHs from other species. The ratios of non-synonymous and synonymous substitution (Ka/Ks) for the paralogs was calculated to test the driving force for duplicated genes; then, we examined the positive selection of SABATH genes using site models and branch-site models under the PAML program. We also analyzed the functional divergence of SABATH genes in *S. miltiorrhiza* using the DIVERGE program. Finally, we analyzed the tissue-specific expression of the SABATH gene family in *S. miltiorrhiza*.

## 2. Materials and Methods

### 2.1. Identification of the Members of SABATH Gene Family in S. miltiorrhiza

The SABATH gene family was first identified in *Arabidopsis*, and twenty-four *AtSABATH* family members have been detected [9]. The nucleotide and amino acids sequences of the 24 *AtSABATH* genes were obtained from the Arabidopsis Information Resource TAIR database (http://www.arabidopsis.org/). Those sequences were then set as queries in a tBLASTn [32] search of the current *S. miltiorrhiza* genome assembly, which covers about 92% of the entire genome and 96% of the protein coding genes [31,33]. An e-value cut-off of 10^−10^ was applied to the homologue recognition. If the sequence satisfied e ≤ 10^−10^, it was selected as a candidate protein. In order to further identify all of the predicted SABATH members in *S. miltiorrhiza*, the Pfam database [34] was used to predict the *SmSABATH* domains of all the candidate proteins. If the SABATH domain was present in the candidate proteins, it belonged to the SABATH gene family of *S. miltiorrhiza*.

### 2.2. Sequence Feature and Gene Structure Analyses

The theoretical isoelectric point (*pI*) and molecular weights (*Mw*) of the SABATH proteins in *S. miltiorrhiza* were predicted using the Compute pI/Mw tool on the ExPASy server (http://web.expasy.org/compute_pi/). The online Gene Structure Display Server (http://gsds1.cbi.pku.edu.cn/) was used to investigate the gene structure, based on each of the coding sequences (CDS) and the corresponding genomic sequences.

### 2.3. Multiple Sequence Alignment, Phylogenetic Analyses and Motif Detection

Multiple sequence alignment of the 30 *SmSABATH* conserved amino acid sequences were performed using the DNAMAN program (Lynnon Corporation, San Ramon, CA, USA). The conserved blocks were obtained using the online Program Gblock 0.91b (http://www.phylogeny.fr/one_task.cgi?task_type=gblocks). An un-rooted tree was constructed using Bayesian inference implemented in MrBayes [35,36], based on the amino acid sequences of the *SmSABATH*, *AtSABATH* and selected known SABATHs from other species (Table S1) under the model of JTT + I + G + F. The model chosen using the program of ProtTest [37]. The phylogenetic tree was represented with the help of Treeview1.61 software [38]. Conserved motifs in *SmSABATH* family were performed by MEME (Suite version 4.9.1: http://alternate.meme-suite.org/tools/meme) with the following criteria: Expected e-values less than 2 × 10^−30^, any number of repetitions of a motif [39,40].

### 2.4. Ka and Ks Calculation

The paralogs for the *SmSABATH* genes were inferred from the phylogenetic tree that was explained in Section 2.3. Non-synonymous (Ka) and synonymous (Ks) substitution rates, and the Ka/Ks ratio of each paralogous gene pair, were determined by PAL2NAL program (http://www.bork.embl.de/pal2nal/) [41]. Meanwhile, the Ka/Ks ratios for all of the paralogous genes were calculated with a sliding window of 20 aa [42].

### 2.5. Tests of Positive Selection

To determine whether the *SmSABATH* gene family exhibited evidence of positive selection under the site models and branch-site models [43], the codeml program in PAML v4.9a was applied to test the hypothesis of positive selection. We reconstructed the phylogenetic tree with the amino acid sequences of *SmSABATH* under the model of JTT + I + G in MrBayes [35,36]. The selected model also used the program ProtTest [37]. In the site model, M0 (one ratio), M3 (discrete), M1a (neutral), M2a (selection), M7 (beta), and M8 (beta & ω) were applied to the alignments, and the variation in the ω parameter among sites were detected using LRT (likelihood ratio test) for M0 vs. M3, M1a vs. M2a, and M7 vs. M8. The branch-site model [44] was used to compare the Ka/Ks ratio between the branches. The positive selection amino acid sites of *SmSABATH* were tested by the improved branch-site model [44]. The branches that were tested for positive selection were used as the foreground, while all of the other branches on the tree were used as the background. Furthermore, the ratio of non-synonymous and synonymous substitution rates for each branch was calculated under the Null Model and Alternative Model. In the Null Model, the omega was set as 1; in the Alternative Model the omega was set as >1. The positive selection sites were detected by comparing the significance level between the Null Model and Alternative Model, using LRT. If LRT suggested the presence of codons under positive selection on the foreground branch, the codon was probably from the site class of positive selection [45]. Posterior probabilities (Qks) were estimated with the Bayes Empirical Bayes (BEB) method [46].

### 2.6. Estimation of Functional Divergence

An analysis of the functional divergence between the *SmSABATH* genes of subgroups was performed using DIVERGE version 3.0 [47]. The method could estimate significant changes in the site-specific shifts, based on maximum likelihood procedures [48]. The estimation was based on the neighbor-joining tree, which was reconstructed with *SmSABATH* amino acid sequences using MEGA 6.0 [49], and the coefficients of Type-I and Type-II functional divergences (*θ*_I_ and *θ*_II_) between two clusters were calculated. The coefficients of Type-I and Type-II functional divergences (*θ*_I_ and *θ*_II_) that were greater than 0 indicated that site-specific altered selective constraints, and a radical shift in amino acid physiochemical properties occurred after gene duplication or speciation [50]. The posterior probabilities (Qks) of amino acid sites that were responsible for functional divergence could also be estimated by this program. A large posterior probability (Qk) represented a high possibility that the evolutionary rate or the radical change in the amino acid property of a site was different between two clusters [50]. Additionally, Qk > 0.8 was empirically used as the cutoff in the identification of Type-I functional divergence-related residues between gene groups. Meanwhile, Type-II functional divergence divergence-related residues were identified with Qk > 1.0 as the cutoff [48].

### 2.7. Plant Materials, RNA Extraction and Real-Time qPCR

Roots, stems, leaves, and flowers were collected from tow-year-old, field-grown *S. miltiorrhiza* Bunge plants from Shangluo County, Shaanxi Province, China, and were stored in liquid nitrogen until use. Total RNA was extracted from the *S. miltiorrhiza* tissues of roots, stems, leaves, and flowers, using the Quick RNA Isolation Kit (Huayueyang, Beijing, China). RNA quantity was determined using a NanoDrop 2000C Spectrophotometer (Thermo Scientific, Wilmington, DE, USA). The first-strand cDNA was synthesized using Prime-Script RT Master Mix (TaKaRa, Beijing, China), according to the manufacture’s protocol. Real-time qPCR was performed using a Light Cycler 96 Instrument (Roche, Basel, Switzerland). The reaction mixture contained 10 μL of 2× SYBR Premix Ex Taq II (Takara, Beijing, China), 20 ng of first-strand cDNA, and 500 nM each of sense and antisense primers. Initial thermal-cycling at 95 °C for 30 s was followed by 45 cycles of 95 °C for 10 s and 60 °C for 30 s. Relative expression was calculated by the 2^−ΔΔ*C*t^ method [51]. All real-time qPCRs were repeated in three biological and three technical replicates. The relative expressions were analyzed as means ± standard deviation (SD). The lengths of the amplicons were between 100 bp and 250 bp. According to Yang et al.’s method [52] for screening the reference genes for real-time qPCR analysis in various tissues of *S. miltiorrhiza*, *Smβ-actin* (DQ243702) was selected as a reference gene. Gene-specific primers were designed using Premier 5.0 and are listed in Table S2.

## 3. Results and Discussion

### 3.1. Sequence Feature of SABATH Genes in S. miltiorrhiza

In order to identify SABATH genes in the *S. miltiorrhiza*, tBLASTn analyses against the *S. miltiorrhiza* genome was performed using *AtSABATH* amino acid sequences as queries. With the BLAST search, a total of 30 *SmSABATH* genes were detected. The gene lengths of *SmSABATH* varied from 1010 bp (*SMil_00022020*) to 5649 bp (*SMil_00022342*) (Table S3), and the lengths of the *SmSABATH* cDNAs and proteins varied from 561 bp and 186 aa (*SMil_00022020*) to 1293 bp and 430 aa (*SMil_00023670*) (Table S3). The molecular weights of the predicted proteins ranged from 20.01 kDa (*SMil_00022020*) to 47.70 kDa (*SMil_00023670*) (Table S3), and the theoretical isoelectric points were predicted to range from 5.03 (*SMil_00021702*) to 9.58 (*SMil_00008156*) (Table S3).

### 3.2. Phylogenetic Analysis of SABATH Gene Family in S. miltiorrhiza

**Figure 1:**
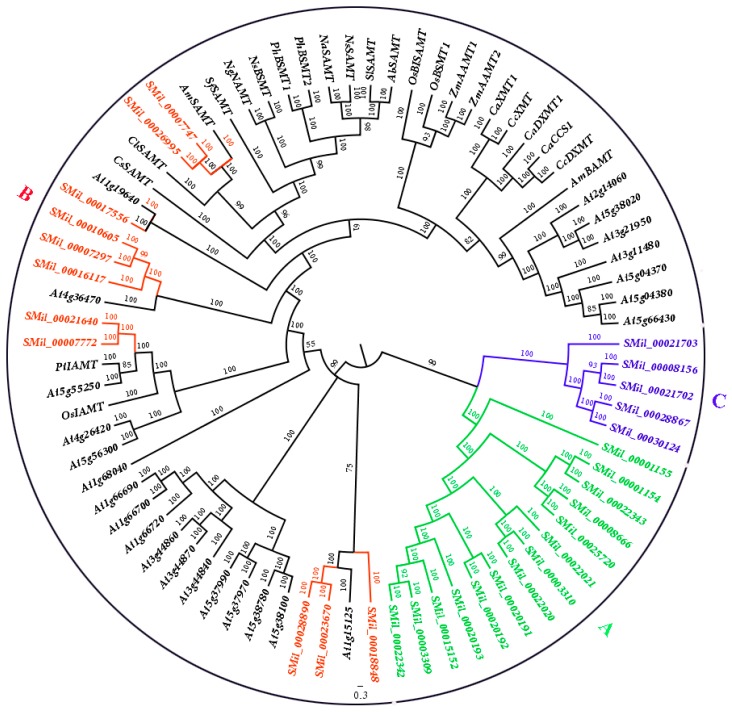
Phylogenetic tree for the SABATH gene family in *Salvia miltiorrhiza* and other species. The tree was constructed using Bayesian inference implemented in MrBayes, based on the amino acid sequences of the *SmSABATH*, *AtSABATH* and other species of the SABATH family under the model of JTT + I + G + F. The members of *SmSABATH* Groups A, B, and C are marked with green, red, and blue, respectively.

To detect the evolutionary relationship of the SABATH gene family in *S. miltiorrhiza* with the members of SABATHs from other species, an un-rooted phylogenetic tree was constructed using the *SmSABATH*, *AtSABATH* and known SABATHs from other species amino acid sequences (Figure 1). Based on the phylogenetic tree, all of the SABATHs were divided into three major groups. The bootstrap values for all of the subgroups were high, suggesting that genes in the same subgroup might share a similar origin (Figure 1). Nearly all of the kindred plant clustered together, and the same function SABATHs were also classed into the same clade (Figure 1). In other words, the SABATHs with higher homology maybe have the same attributes and same function. Thus, it suggested that we could infer the function of unknown SABATHs according to the clustering situation.

For the SABATHs in *S. miltiorrhiza*, all of the *SmSABATH* genes were also divided into three major groups. The fourteen and five *SmSABATHs* clustered together (Group A and Group C), while the other eleven *SmSABATHs* were divided among other species (Group B) (Figure 1). This suggested that the members in Group B may be divergent with other groups in function. Another main objective of this phylogenetic study was to identify putative orthologous and paralogous. Paralogs usually display different functions while orthologs may retain the same function [53,54]. According to the phylogenetic tree (Figure 1), among the 30 *SmSABATH* genes, 11 pairs of paralogous were identified from the SABATH gene family in *S. miltiorrhiza* including *SMil_00003309* and *SMil_00022342*, *SMil_00020191* and *SMil_00020192*, *SMil_00003310* and *SMil_00022020*, *SMil_00008666* and *SMil_00025720*, *SMil_00001154* and *SMil_00022343* in Group A; *SMil_00007297* and *SMil_00010605*, *SMil_00007747* and *SMil_00026995*, *SMil_00007772* and *SMil_00021640*, *SMil_00023670* and *SMil_00028890* in Group B; *SMil_00008156* and *SMil_00021702*, *SMil_00028867,* and *SMil_00030124* in Group C (Figure 1). In addition, one pairs of orthologs genes of *SMil_00017556* and *At1g19640* regarding to the SABATH gene family in *S. miltiorrhiza* were identified (Figure 1), which may have the same function.

### 3.3. Gene Structure Analysis of SABATH Genes in S. miltiorrhiza

**Figure 2:**
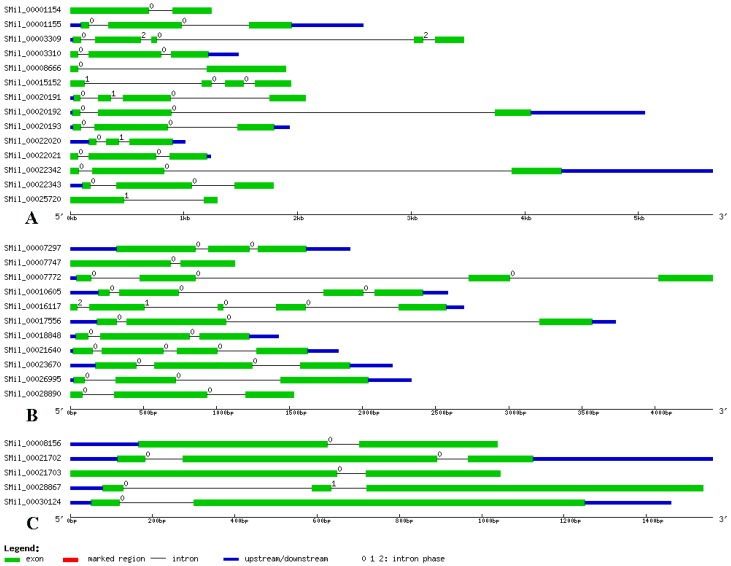
The structural features of each SABATH gene in *S. miltiorrhiza*. The exons are shown using green rectangles, while black lines connecting two exons represent introns. The numbers above the line represent the intron phase.

The structural features of each of the SABATH genes in groups are shown in Figure 2. Structural analyses of all of the SABATH genes in *S. miltiorrhiza* revealed that the number of exons varied from two to five. There were no intronless genes. The average exon numbers in the groups were two or three. We also found that genes in the same group had similar gene structures (Figure 2), which indicated that the genes in same group may have similar functions. Otherwise, symmetric exons were the exons that have the same splicing phase at both ends. The excess of symmetric exons and phase 0 introns are likely to facilitate exon shuffling, recombinational fusion, and protein domain exchange [55,56]. According to the 92 exons analyzed herein, 22 exons were symmetric with phase 0 introns, and no exons were symmetric with phase 1 and 2 introns. Among the 62 introns of the SABATH genes, 53 were phase 0, six were phase 1, and three were phase 2. Therefore, our analyses of the gene structures indicated diversity amongst the SABATH genes in *S. miltiorrhiza*.

### 3.4. Analysis of Conserved Domains and Motifs

**Figure 3:**
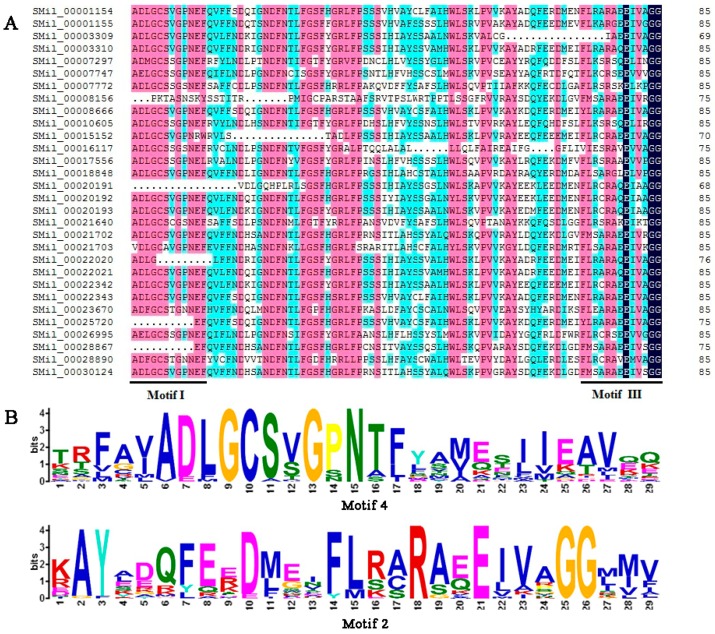
(**A**) Multiple sequence alignments of the *SmSABATH* proteins’ conserved domain of *O*-methyltransferases, including the binding sites (motifs I and III were indicated) of *S*-adenosyl-l-methionine. (**B**) Sequence logo of two conserved motifs; motif 4 and motif 2.

The SABATH domains that are contained in all of the deduced SABATH proteins in *S. miltiorrhiza* were identified using the Pfam program, and the results showed that all members of the *SmSABATH* gene family contained a conserved domain, which was conserved among *O*-methyltransferases [57,58]. Then, the conserved domain sequences were aligned using software DNAMAN; most members of *SmSABATH* proteins contained binding sites (the motifs I and III) of SAM (*S*-adenosyl-l-methionine) (Figure 3), a well-known methyl donor in plant cells [11]. Using the MEME program, we identified 13 conserved motifs in *SmSABATH* amino acid sequences (Table S4). The lengths of the motifs varied from 14 to 50 amino acids, and the number of motifs in each *SmSABATH* ranged from 4 to 11. The frequency of all 13 conserved motifs in *SmSABATH* proteins varied from 7 to 28 (Table S4). Some motifs, such as motifs 1, 2, 3, 4, and 8, were diffusely distributed among *SmSABATH* proteins (Table S5). Among the 13 conserved motifs, motifs 1, 2, 3 and 4 were located in the *SmSABATH* conserved domain, while the other nine motifs were located outside the conserved domain. Otherwise, motif 4 matched motif I while motif 2 matched motif III (Figure 3). Based on the groups indicated by the phylogenetic tree, most *SmSABATH* proteins in the same group had similar motifs compositions and the orders of the motifs were very similar in each group (Table S5), which suggested that the proteins in the same group might have similar functions in plant development. Some specific motifs were located in the proteins of specific groups; for instance, motif 7 was specific to Groups A and B (Table S5). This indicated that the *SmSABATH* proteins may exhibit functional divergence in different groups.

### 3.5. Driving Forces for Genetic Divergence

**Figure 4:**
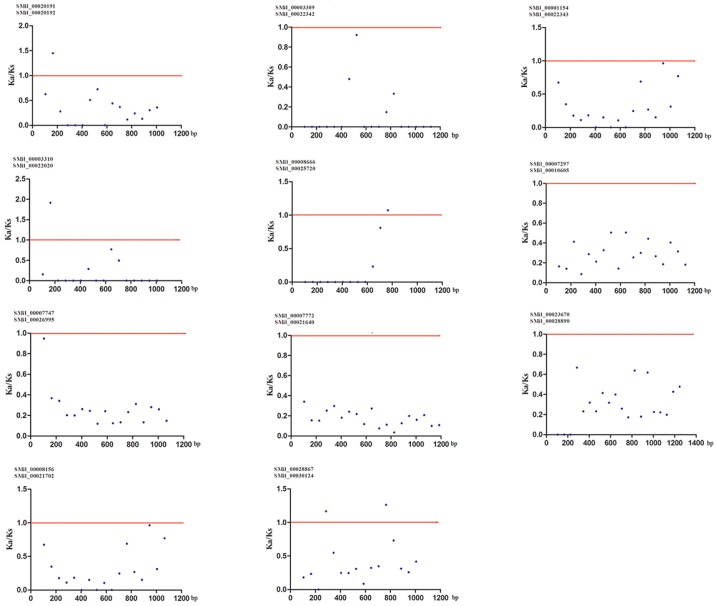
Ka (non-synonymous)/Ks (synonymous) ratios for 11 SmSABATH paralogous pairs of proteins with a sliding window of 20 amino acids. The plot shows the Ka/Ks ratios at various positions for the coding region of SmSABATH genes.

Gene duplication is an important event for gene family expansion and functional diversity during evolution [59]. To detect whether Darwinian positive selection was involved in the driving of gene divergence after duplication, the Ka/Ks ratios of non-synonymous and synonymous substitution rates were calculated using the CDS of paralogous *SmSABATH*. Generally, a Ka/Ks ratio < 1 indicates a negative or purifying selection, a ratio = 1 indicates a neutral evolution and a ratio > 1 indicates a positive selection [60]. According to the paralogous genes determined from the phylogenetic tree, there were 11 pairs of paralogs in *SmSABATH* genes (Figure 1). In other words, more than 73% of *SmSABATH* genes appeared to be duplicated. This suggested that most *SmSABATH* genes have happened functional diversity and gene family expansion during evolution. The Ka/Ks ratios for all of the 11 *SmSABATH* paralogous pairs were <1 (Table S6), suggesting that the *SmSABATH* genes have experienced purifying selection pressure. Meanwhile, we also calculated the Ka/Ks ratios for all of the paralogous genes with a sliding window of 20 aa. The ratios of Ka/Ks that were higher than 1 in the regions of all paralogous genes indicated that these regions had gone through positive selections. However, the proportion of such regions were few. Most of the regions showed that the ratios of Ka/Ks were less than 1 in paralogous genes, and this also indicated that the SABATH genes in *S. miltiorrhiza* had undergone purifying selection (Figure 4).

### 3.6. Positive Selection on SmSABATH Genes

In order to preliminarily examine the evolutionary mechanisms of the *SmSABATH* gene family, we tested the hypothesis of positive selection of the genes using site models and branch-site models in PAML program [43,46] base on the phylogenetic tree (Figure S1).

In site models, there were six codon substitution models [43], including M0, M1a, M2a, M3, M7, and M8, which were applied to the alignments and these models assume variation in ω among sites. The results of these models’ parameter estimates, log likelihood, and the LRT tests are shown in Table 1. To examine how dN/dS (non-synonymous/synonymous) ratios differed among codon positions, models M0 and M3 were compared. M0 (one ratio) assumes that the different sites have the same evolution rate, while M3 (discrete) assumes a general discrete distribution with three site classes (*p*_0_, *p*_1_, *p*_2_) [48]. The log likelihood of M0 for *SmSABATH* sequences was ι = −24,100.940606, with an estimate of ω = 0.32227. The log likelihood of M3 was ι = −23,631.868500, with an estimate of ω_0_ = 0.06316, ω_1_ = 0.25310, and ω_2_ = 0.65803 (Table 1). These results indicated that all of the codons were under purifying selection. Additionally, the value of twice the log likelihood difference (2ΔlnL) between M3 and M0 was 938.1442, which was strongly statistically significant (*p* < 0.01) and suggested that M3 was better than M0. Therefore, the results indicated that different sites bare different selection pressures and also indicated fluctuations in the overall level of selective constraints.

The codon substitution models of M2a and M8 allow for positive selection, while the models of M1a and M7 hypothesize a nearly neutral selection [48]. We could test whether positive selection promoted divergence between genes through comparing the M2a vs. M1a and M8 vs. M7, respectively. The log likelihood of M1a and M2a for *SmSABATH* genes was ι = −23,766.855086. The value of 2ΔlnL between M1a and M2a was 0; it was not statistically significant, and no sites were positively selected at a level of 95% (Table 1). The log likelihood of M7 and M8 for *SmSABATH* genes was ι = −23,622.773935 and ι = −23,622.775486, respectively. The value of 2ΔlnL between M7 and M8 was close to 0 (Table 1); it was also not statistically significant. Although six sites were detected, they were not positively selected at a level of 95% (Table 1). In both cases, no significant evidence of positive selection was found.

Branch-site models were designed to detect positive selection that was affecting a few sites along particular lineages, and allowed ω ratios to simultaneously vary among sites and branches [46]. The parameter estimates for branches under positive selection are list in Table 2. When Group A was set as the foreground branch, the value of 2ΔlnL between the Null and Alternative models was close to 0, and no positive sites were found (Table 2). In addition, three sites were found when Group B was set as the foreground branch, but they were not positively selected at a level of 95%, and the value of 2ΔlnL between Null and Alternative models was 5.653506 (*p* = 0.017) (Table 2). When Group C was set as the foreground branch, the value of 2ΔlnL between Null and Alternative models was 12.07159 (*p* < 0.01) (Table 2). A total of four sites were found but only one site in Group C was positively selected at a level of 95% (Table 2). In other words, only one positive site and one lineage group were found to be under positive selection. Moreover, the results suggested that different *SmSABATH* lineages may have different evolutionary rates. Group A evolution seemed to be more conservative; Group B might be confronted with positive Darwinian selection, but no positive sites were detected; and, Group C could be confronted with strong positive Darwinian selection, since significant positive sites were detected at the 0.05 significance level (Table 2).

### 3.7. Functional Divergence Analysis (FDA) of SmSABATH Proteins

With the program DIVERGE 3.0, the shifted evolutionary rates and altered amino acid properties after gene duplication could be evaluated [50,61]. The Type-I functional divergence (*θ*_I_) was based on evolutionary rate [50] and the Type-II functional divergence (*θ*_II_) was based on differences in the biochemical properties of amino acids [61]. According to the neighbor-joining tree, the *SmSABATH* amino acid sequences were also divided into three major clusters (Cluster A, Cluster B, and Cluster C) (Figure S2). Posterior probability (Qk) was carried out to estimate Type-I and Type-II between the *SmSABATH* clusters.

Using DIVERGE, we found that all of the coefficients for Type-I functional divergence (*θ*_I_) were greater than zero in the three group pairs, Group A vs. Group B, Group A vs. Group C, and Group B vs. Group C, and ranged from 0.286372 to 0.683167 (Table S7). This suggested that certain amino acid sites may have experienced significant site-specific changes between these group pairs, leading to a subgroup-specific functional evolution after their diversification. We also found that Type-I functional divergence (*θ*_I_) of Group A vs. Group B and Group B vs. Group C were statistically significant (*p* = 0.016727 and *p* = 0.015877, respectively) (Table S7), while Group A vs. Group C was not statistically significant (*p* = 0.198275). This indicated that the members in Group B may be different from those of other groups in terms of function. This also verifies the phylogenetic analyses that the members in Group B have divergent with those of other groups in function. Some residues in this group probably play important roles in the functional divergence of *SmSABATH* genes. The coefficients for Type-II functional divergence (*θ*_II_) in two group pairs, Group A vs. Group B, Group A vs. Group C, were greater than zero (0.041610 and 0.117169, respectively), which indicated a radical shift in amino acid properties, while the coefficients in Group B vs. Group C was less than zero (Table S8). However, the Type-II coefficients were not statistically significant among the three group pairs (*p* = 0.468158, *p* = 0.370624, and *p* = 0.312775, respectively) (Table S8), which suggested that most residues in the *SmSABATH* family should not experience obvious physical and chemical property changes.

**Figure 5:**
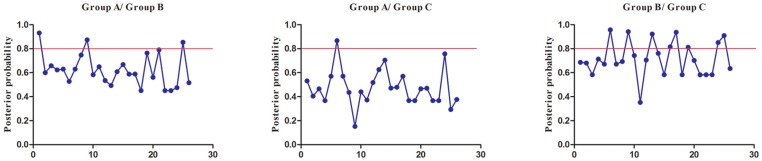
Site-specific prediction for type-I functional divergence between groups of *SmSABATH*. The X-axis represents the locations of sites. The Y-axis represents the probabilities of each group. The red line indicates a cutoff = 0.80.

**Figure 6:**
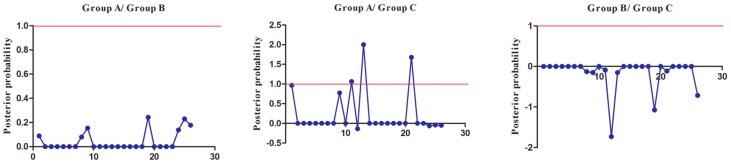
Site-specific profile for predicting critical amino acid residues responsible for type-II functional divergence between groups of *SmSABATH*. The X-axis represents the locations of sites. The Y-axis represents the probabilities of each group. The red line indicates a cutoff = 1.0.

To extensively reduce positive false, we used Qk > 0.8 and 1.0 as the cutoff to identify Type-I and Type-II functional divergence-related positive selection sites between gene groups, respectively. Using the detailed posterior probabilities analysis, we found that the distribution and the number of positive selection sites for functional divergence in group pairs were different. In Type-I functional divergence, when Qk > 0.8, all three of the group pairs contained positive selection sites (Table S7). Meanwhile, in Type-II functional divergence, when Qk > 1.0, we found that only one group pair (Group A vs. Group C) contained three positive selection sites. No positive selection sites were found in other tow group pairs (Table S8). This suggested that these sites probably play an important part in the functional divergence of *SmSABATH* during the evolutionary process. A detailed distribution of site-specific predictions for Type-I and Type-II functional divergence of *SmSABATH* between groups are showed in Figure 5 and Figure 6.

### 3.8. Tissue-Specific Expression of SABATH Gene Family in S. miltiorrhiza

**Figure 7:**
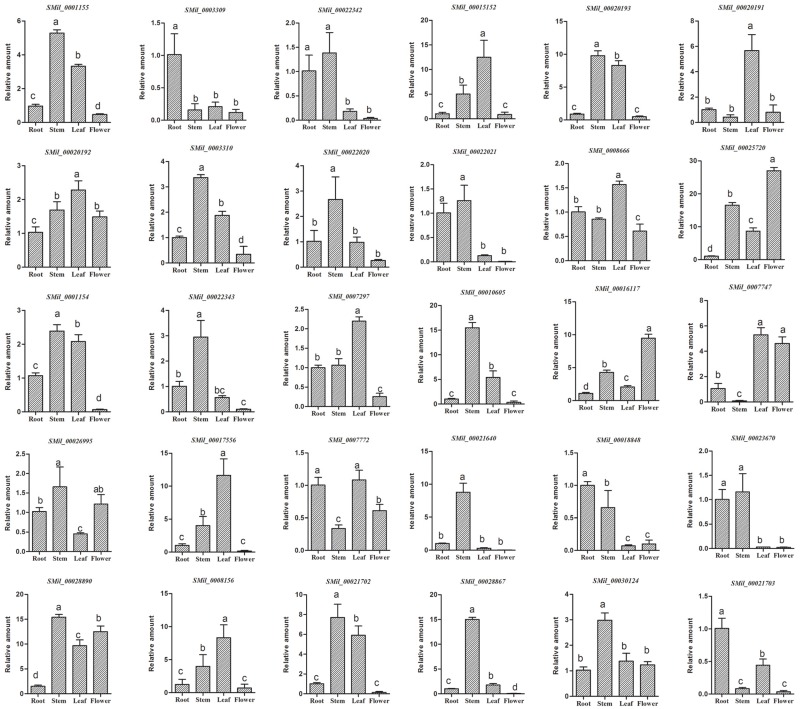
Tissue-specific expression of the SABATH gene family in *S. miltiorrhiza*. The expression level of *SmSABATHs* was analyzed by the 2^−ΔΔ*C*t^ method. Y-axis indicates the relative expression levels. X-axis indicates different tissues. Transcript levels in roots were arbitrarily set to 1, and the levels in other tissues were determined relative to this. Error bars represent standard deviations of mean value from three biological and three technical replicates. Analysis of variance was calculated using SPSS. *p* < 0.05 was considered to be statistically significant.

In order to preliminarily detect the tissue-specific expression of the SABATH gene family in *S. miltiorrhiza*, we analyzed the expression of *SmSABATH* in the roots, stems, leaves, and flowers of *S. miltiorrhiza* plants. All of the 30 *SmSABATHs* that were identified exhibited differential expression patterns (Figure 7). Among the 30 *SmSABATHs*, three (10.0%) showed predominant expression in roots, seven (23.3%) mainly expressed in leaves, 13 (43.3%) were primarily expressed in stems, and two (6.7%) were in flowers. In addition, five (16.7%) *SmSABATHs* were mainly expressed in at least two tissues analyzed, indicating that these genes may play a more ubiquitous role in *S. miltiorrhiza*. On the whole, the SABATH gene family in *S. miltiorrhiza* was primarily expressed in stems and leaves. This suggested that the SABATH gene family is related to the plant defense against insects that gnaw at the leaves and stems of plants. Once a plant is damaged from the outside environment, the products of SABATH genes, such as methyl jasmonate or methyl salicylate, can enhance the level of plant resistance [11].

## 4. Conclusions

SABATH genes are ubiquitous in higher plants and play important roles in various processes of plant development. Further studies on this family could not only illustrate the SABATH genes’ vital functions in the developmental processes of higher plants, but also elucidate the evolutionary relationships between different species. In this study, we identified 30 members of SABATH genes in the *S. miltiorrhiza* genome database. The identified *SmSABATH* genes were characterized using a comprehensive approach, including gene structure analyses, SABATH domain characterization, phylogenetic analyses, conserved motif identification, positive selection analyses, functional divergence analyses, and tissue-specific expression. We showed that 30 *SmSABATH* genes could be divided into three groups, according to the phylogenetic tree. SABATH genes in *S. miltiorrhiza* have experienced strong purifying selection pressures, since the Ka/Ks ratios for all 11 paralogous were <1. A total of 13 conserved motifs were identified, of which some group-specific motifs might be attributable to the functional divergence of SABATH genes in *S. miltiorrhiza*. Functional divergence analyses also showed that the *SmSABATH* genes have diverged in terms of function. Positive selection analyses with site model and branch-site model showed that SABATH genes in *S. miltiorrhiza* experienced positive selection. Tissue-specific expression showed that the SABATH gene family in *S. miltiorrhiza* is primarily expressed in stems and leaves. These results provide abundant information regarding *SmSABATH* and are useful in further studying SABATH gene functions in *S. miltiorrhiza*.

## Figures and Tables

**Table 1:** Tests for positive selection among codons of *SmSABATH* genes using site models.

Model	np	lnL	Estimates of Parameter ^1^		2ΔlnL	Positive Selection Sites ^2^
			Frequency	dN/dS		
M0 (one-ratio)	60	−24,100.940606		0.32227		None
M3 (discrete)	64	−23,631.868500	*p*_0_ = 0.15469 *p*_1_ = 0.41191 *p*_2_ = 0.43340	ω_0_ = 0.06316 ω_1_ = 0.25310 ω_2_ = 0.65803	938.1442 (M3 vs. M0) **	Not allowed
M1a (nearly neutral)	61	−23,766.855086	*p*_0_ = 0.53255 *p*_1_ = 0.46745	ω_0_ = 0.23043 ω_1_ = 1.00000		None
M2a (positive selection)	63	−23,766.855086	*p*_0_ = 0.53255 *p*_1_ = 0.30714 *p*_2_ = 0.16031	ω_0_ = 0.23043 ω_1_ = 1.00000 ω_2_ = 1.00000	0 (M2a vs. M1a)	Not allowed
M7 (beta)	61	−23,622.773935	*p* = 1.10441 *q* = 1.67064			None
M8 (beta & ω)	63	−23,622.775486	*p*_0_ = 0.99999 *p* = 1.10448 *q* = 1.67080 *p*_1_ = 0.00001	ω = 5.70606	0.00308 (M8 vs. M7)	181 Q 432 V 434 G 460 S 476 H 479 C

Note: * *p* < 0.05 and ** *p* < 0.01 (*x*^2^ test). ^1^ ω was estimated under models; *p* and *q* were the parameters of the beta distribution. ^2^ The number of amino acid sites estimated to have undergone positive selection. np was the number of parameter. lnL was the values of log-likelihood. dN/dS was non-synonymous/synonymous. 2ΔlnL was the value of twice the log likelihood difference between models.

**Table 2:** Selective pressure analyses of SABATH genes in *S. miltiorrhiza* by branch-site model.

Foreground Branches	Branch-Site Model	lnL	2ΔlnL	*p* Value	ω Values ^1^	Positively Selected Sites ^2^
Group A	Null	−23,766.855109	0	1	ω_0_ = 0.23043 ω_1_ = 1.00000 ω_2_ = 1.00000	None
	Alternative	−23,766.855151			ω_0_ = 0.23043 ω_1_ = 1.00000 ω_2_ = 3.05353	
Group B	Null	−23,766.855088	5.653506	0.017	ω_0_ = 0.23043 ω_1_ = 1.00000 ω_2_ = 1.00000	152 A 0.649 209 A 0.815 385 R 0.516
	Alternative	−23,764.028335			ω_0_ = 0.23051 ω_1_ = 1.00000 ω_2_ = 464.10117	
Group C	Null	−23,766.466691	12.07159	<0.01	ω_0_ = 0.22946 ω_1_ = 1.00000 ω_2_ = 1.00000	137 K 0.772 200 I 0.514 252 H 0.889 461 A 0.961 *
	Alternative	−23,760.430894			ω_0_ = 0.22951 ω_1_ = 1.00000 ω_2_ = 998.97795	

Note: * *p* < 0.05 and ** *p* < 0.01 (x^2^ test). ^1^ ω was estimated under model Null and Alternative. ^2^ The number of amino acid sites estimated to have undergone positive selection. lnL was the values of log-likelihood. 2ΔlnL was the value of twice the log likelihood difference between models.

## Supplementary Materials

The following are available online at www.mdpi.com/2073-4425/8/12/365/s1. Table S1: List of the SABATH genes from other species. Table S2: Primers for qRT-PCR. Table S3: Gene features of *SmSABATH*. Table S4: Normal expression sequences of 13 motifs identified in 30 *SmSABATH* proteins. Table S5: The SABATH protein motif diagram of *S. miltiorrhiza*. Table S6: Ka/Ks and divergence analysis of SABATH paralogous in *S. miltiorrhiza*. Table S7: The coefficient of Type-I functional divergence (*θ*_I_) from pairwise comparisons between *SmSABATH* groups. Table S8: The coefficient of Type-II functional divergence (*θ*_II_) from pairwise comparisons between *SmSABATH* groups. Figure S1. Phylogenetic tree was reconstructed using the Bayesian inference method under the JTT + I + G model with the *SmSABATH* amino acid sequences. Figure S2. The neighbor-joining phylogenetic tree was reconstructed with *SmSABATH* amino acid sequences using MEGA 6.0.

## References

[1] Attieh J., Djiana R., Koonjul P., Étienne C., Sparace S.A., Saini H.S. (2002). Cloning and functional expression of two plant thiol methyltransferases: A new class of enzymes involved in the biosynthesis of sulfur volatiles. Plant Mol. Biol..

[2] Effmert U., Saschenbrecker S., Ross J., Negre F., Fraser C.M., Noel J.P., Dudareva N., Piechulla B. (2005). Floral benzenoid carboxyl methyltransferases: From in vitro to in planta function. Phytochemistry.

[3] Zubieta C. (2002). Structural basis for the modulation of lignin monomer methylation by caffeic Acid/5-Hydroxyferulic Acid 3/5-*O*-Methyltransferase. Plant Cell Online.

[4] Zubieta C. (2003). Structural basis for substrate recognition in the salicylic acid carboxyl methyltransferase family. Plant Cell Online.

[5] Zubieta C., He X.Z., Dixon R.A., Noel J.P. (2001). Structures of two natural product methyltransferases reveal the basis for substrate specificity in plant *O*-methyltransferases. Nat. Struct. Biol..

[6] Noel J.P., Dixon R.A., Pichersky E., Zubieta C., Ferrer J.L. (2003). Chapter two Structural, functional, and evolutionary basis for methylation of plant small molecules. Recent Adv. Phytochem..

[7] Ross J.R., Nam K.H., D’Auria J.C., Pichersky E. (1999). *S*-adenosyl-l-methionine: Salicylic acid carboxyl methyltransferase, an enzyme involved in floral scent production and plant defense, represents a new class of plant methyltransferases. Arch. Biochem. Biophys..

[8] Murfitt L.M., Kolosova N., Mann C.J., Dudareva N. (2000). Purification and characterization of *S*-adenosyl-l-methionine: Benzoic acid carboxyl methyltransferase, the enzyme responsible for biosynthesis of the volatile ester methyl benzoate in flowers of *Antirrhinum majus*. Arch. Biochem. Biophys..

[9] D’Auria J.C., Chen F., Pichersky E. (2003). Chapter eleven the SABATH family of MTS in *Arabidopsis thaliana* and other plant species. Recent Adv. Phytochem..

[10] Zhao N., Ferrer J.L., Moon H.S., Kapteyn J., Zhuang X., Hasebe M., Stewart C.N., Gang D.R., Chen F. (2012). A SABATH Methyltransferase from the moss *Physcomitrella patens* catalyzes *S*-methylation of thiols and has a role in detoxification. Phytochemistry.

[11] Seo H.S., Song J.T., Cheong J.J., Lee Y.H., Lee Y.W., Hwang I., Lee J.S., Choi Y.D. (2001). Jasmonic acid carboxyl methyltransferase: A key enzyme for jasmonate-regulated plant responses. Proc. Natl. Acad. Sci. USA.

[12] Qin G., Gu H., Zhao Y., Ma Z., Shi G., Yang Y., Pichersky E., Chen H., Liu M., Chen Z. (2005). An indole-3-acetic acid carboxyl methyltransferase regulates *Arabidopsis* leaf development. Plant Cell.

[13] Yang Y., Yuan J.S., Ross J., Noel J.P., Pichersky E., Chen F. (2006). An *Arabidopsis thaliana* methyltransferase capable of methylating farnesoic acid. Arch. Biochem. Biophys..

[14] Varbanova M., Yamaguchi S., Yang Y., McKelvey K., Hanada A., Borochov R., Yu F., Jikumaru Y., Ross J., Cortes D. (2007). Methylation of gibberellins by *Arabidopsis* GAMT_1_ and GAMT_2_. Plant Cell.

[15] Kapteyn J., Qualley A.V., Xie Z., Fridman E., Dudareva N., Gang D.R. (2007). Evolution of cinnamate/*p*-coumarate carboxyl methyltransferases and their role in the biosynthesis of methylcinnamate. Plant Cell.

[16] Murata J., Roepke J., Gordon H., De Luca V. (2008). The leaf epidermome of *Catharanthus roseus* reveals its biochemical specialization. Plant Cell.

[17] Kollner T.G., Lenk C., Zhao N., Seidl-Adams I., Gershenzon J., Chen F., Degenhardt J. (2010). Herbivore-induced SABATH methyltransferases of maize that methylate anthranilic acid using *S*-adenosyl-l-methionine. Plant Physiol..

[18] Ogawa M., Herai Y., Koizumi N., Kusano T., Sano H. (2000). 7-Methylxanthine methyltransferase of coffee plants. Gene isolation and enzymatic properties. J. Biol. Chem..

[19] Zhao N., Boyle B., Duval I., Ferrer J.L., Lin H., Seguin A., MacKay J., Chen F. (2009). SABATH methyltransferases from white spruce (*Picea glauca*): Gene cloning, functional characterization and structural analysis. Tree Physiol..

[20] And R.A.C., Mullet J.E. (1997). Biosynthesis and action of jasmonates in plants. Ann. Rev. Plant Physiol. Plant Mol. Biol..

[21] Wasternack C., Hause B. (2002). Jasmonates and octadecanoids: Signals in plant stress responses and development. Prog. Nucleic Acid Res. Mol. Biol..

[22] Ljung K., Hull A.K., Kowalczyk M., Marchant A., Celenza J., Cohen J.D., Sandberg G. (2002). Biosynthesis, conjugation, catabolism and homeostasis of indole-3-acetic acid in *Arabidopsis thaliana*. Auxin Molecular Biology.

[23] Woodward A.W., Bartel B. (2005). Auxin: Regulation, action, and interaction. Ann. Bot..

[24] Chen F., D’Auria J.C., Tholl D., Ross J.R., Gershenzon J., Noel J.P., Pichersky E. (2003). An *Arabidopsis thaliana* gene for methylsalicylate biosynthesis, identified by a biochemical genomics approach, has a role in defense. Plant J. Cell Mol. Biol..

[25] Xu R., Song F., Zheng Z. (2006). *OsBISAMT*_1_, a gene encoding *S*-adenosyl-l-methionine: Salicylic acid carboxyl methyltransferase, is differentially expressed in rice defense responses. Mol. Biol. Rep..

[26] Uefuji H., Tatsumi Y., Morimoto M., Kaothien-Nakayama P., Ogita S., Sano H. (2005). Caffeine production in tobacco plants by simultaneous expression of three coffee *N*-methyltrasferases and its potential as a pest repellant. Plant Mol. Biol..

[27] Seifert K., Unger W. (1994). Insecticidal and fungicidal compounds from *Isatis tinctoria*. Z. Naturforsch. C J. Biosci..

[28] Li Y.G., Song L., Liu M., Hu Z.B., Wang Z.T. (2009). Advancement in analysis of *Salviae miltiorrhizae* Radix et Rhizoma (Danshen). J. Chromatogr. A.

[29] Ma Y., Yuan L., Wu B., Li X., Chen S., Lu S. (2012). Genome-wide identification and characterization of novel genes involved in terpenoid biosynthesis in *Salvia miltiorrhiza*. J. Exp. Bot..

[30] Wang Q.H., Chen A.H., Zhang B.L. (2009). Salviae Miltiorrhiza: A model organism for chinese traditional medicine genomic studies. Acta Chin. Med. Pharmacol..

[31] Xu H., Song J., Luo H., Zhang Y., Li Q., Zhu Y., Xu J., Li Y., Song C., Wang B. (2016). Analysis of the genome sequence of the medicinal plant *Salvia miltiorrhiza*. Mol. Plant.

[32] Altschul S.F., Madden T.L., Schäffer A.A., Zhang J., Zhang Z., Miller W., Lipman D.J. (1997). Gapped BLAST and PSI-BLAST: A new generation of protein database search programs. Nucleic Acids Res..

[33] Song J.Y., Luo H.M., Li C.F., Sun C., Xu J., Chen S.L. (2013). *Salvia miltiorrhiza* as medicinal model plant. Acta Pharm. Sin..

[34] Bateman A., Coin L., Durbin R., Finn R.D., Hollich V., Griffithsjones S., Khanna A., Marshall M., Moxon S., Sonnhammer E.L. (2004). The Pfam protein families database. Nucleic Acids Res..

[35] Huelsenbeck J.P., Ronquist F. (2001). MRBAYES: Bayesian inference of phylogenetic trees. Bioinformatics.

[36] Hall B.G. (2005). Comparison of the accuracies of several phylogenetic methods using protein and DNA sequences. Mol. Biol. Evol..

[37] Abascal F., Zardoya R., Posada D. (2005). ProtTest: Selection of best-fit models of protein evolution. Bioinformatics.

[38] Zhai Y., Tchieu J., Saier S.M. (2002). A web-based Tree View (TV) program for the visualization of phylogenetic trees. J. Mol. Microbiol. Biotechnol..

[39] Bailey T.L., Williams N., Misleh C., Li W.W. (2006). MEME: Discovering and analyzing DNA and protein sequence motifs. Nucleic Acids Res..

[40] Zhang X., Luo H., Xu Z., Zhu Y., Ji A., Song J., Chen S. (2015). Genome-wide characterisation and analysis of bHLH transcription factors related to tanshinone biosynthesis in *Salvia miltiorrhiza*. Sci. Rep..

[41] Suyama M., Torrents D., Bork P. (2006). PAL2NAL: Robust conversion of protein sequence alignments into the corresponding codon alignments. Nucleic Acids Res..

[42] Fares M.A. (2004). SWAPSC: Sliding window analysis procedure to detect selective constraints. Bioinformatics.

[43] Yang Z., Nielsen R., Goldman N., Pedersen A.M. (2000). Codon-substitution models for heterogeneous selection pressure at amino acid sites. Genetics.

[44] Zhang J., Nielsen R., Yang Z. (2005). Evaluation of an improved branch-site likelihood method for detecting positive selection at the molecular level. Mol. Biol. Evol..

[45] Yang Z., Wong W.S., Nielsen R. (2005). Bayes empirical bayes inference of amino acid sites under positive selection. Mol. Biol. Evol..

[46] Yang Z. (2007). PAML 4: Phylogenetic analysis by maximum likelihood. Mol. Biol. Evol..

[47] Gu X., Zou Y., Su Z., Huang W., Zhou Z., Arendsee Z., Zeng Y. (2013). An update of DIVERGE software for functional divergence analysis of protein family. Mol. Biol. Evol..

[48] Li C., Li D., Shao F., Lu S. (2015). Molecular cloning and expression analysis of WRKY transcription factor genes in *Salvia miltiorrhiza*. BMC Genom..

[49] Tamura K., Stecher G., Peterson D., Filipski A., Kumar S. (2013). MEGA6: Molecular Evolutionary Genetics Analysis Version 6.0. Mol. Biol. Evol..

[50] Gu X. (1999). Statistical methods for testing functional divergence after gene duplication. Mol. Biol. Evol..

[51] Schmittgen T.D., Livak K.J. (2008). Analyzing real-time PCR data by the comparative Ct method. Nat. Protoc..

[52] Yang Y., Hou S., Cui G., Chen S., Wei J., Huang L. (2010). Characterization of reference genes for quantitative real-time PCR analysis in various tissues of *Salvia miltiorrhiza*. Mol. Biol. Rep..

[53] Yang Z., Wang X., Gu S., Hu Z., Xu H., Xu C. (2008). Comparative study of SBP-box gene family in *Arabidopsis* and rice. Gene.

[54] Yang Z., Zhou Y., Wang X., Gu S., Yu J., Liang G., Yan C., Xu C. (2008). Genomewide comparative phylogenetic and molecular evolutionary analysis of tubby-like protein family in *Arabidopsis*, rice, and poplar. Genomics.

[55] Gilbert W. (1987). The exon theory of genes. Cold Spring Harb. Symp. Quant. Biol..

[56] Patthy L. (1987). Intron-dependent evolution: Preferred types of exons and introns. FEBS Lett..

[57] Joshi C.P., Chiang V.L. (1998). Conserved sequence motifs in plant *S*-adenosyl-l-methionine-dependent methyltransferases. Plant Mol. Biol..

[58] Kagan R.M., Clarke S. (1994). Widespread occurrence of three sequence motifs in diverse *S*-adenosylmethionine-dependent methyltransferases suggests a common structure for these enzymes. Arch. Biochem. Biophys..

[59] Lynch M., Conery J.S. (2000). The evolutionary fate and consequences of duplicate genes. Science.

[60] Wang W., Zheng H., Yang S., Yu H., Li J., Jiang H., Su J., Yang L., Zhang J., Mcdermott J. (2005). Origin and evolution of new exons in rodents. Genome Res..

[61] Gu X. (2006). A simple statistical method for estimating type-II (cluster-specific) functional divergence of protein sequences. Mol. Biol. Evol..

