# Supplementary material for: Genome-Wide Comprehensive Analysis the Molecular Phylogenetic Evaluation and Tissue-Specific Expression of SABATH Gene Family in Salvia miltiorrhiza
Source: Genes (Basel). 2017 Dec 5;8(12):365. doi: 10.3390/genes8120365 (PMC5748683; doi:10.3390/genes8120365)
Supplement: Supplementary file 1 [file genes-08-00365-s001.zip › Supplementary File(s)/Supplementary caption.docx]

**Table S1**:List of the SABATH genes from other species

**Table S2:** Primers for qRT-PCR

**Table S3:** Gene features of *SmSABATH*

**Table S4:** Normal expression sequences of 13 motifs identified in 30 *SmSABATH* proteins

**Table S5:** The SABATH protein motif diagram of *S.miltiorrhiza*

**Table S6:** Ka/Ks and divergence analysis of SABATH paralogous in *S.miltiorrhiza*

**Table S7:** The coefficient of Type-I functional divergence (*θ*_I_) from pairwise comparisons between *SmSABATH* groups

**Table S8:** The coefficient of Type-Ⅱfunctional divergence (*θ*_Ⅱ_) from pairwise comparisons between *SmSABATH* groups

**Figure S1**. Phylogenetic tree was reconstructed using the Bayesian inference method under the JTT+I+G model with the *SmSABATH* amino acid sequences

**Figure S2**. The neighbor-joining phylogenetic tree was reconstructed with *SmSABATH* amino acid sequences using MEGA 6.0.
