# Supplementary material for: Genome-Wide Comprehensive Analysis the Molecular Phylogenetic Evaluation and Tissue-Specific Expression of SABATH Gene Family in Salvia miltiorrhiza
Source: Genes (Basel). 2017 Dec 5;8(12):365. doi: 10.3390/genes8120365 (PMC5748683; doi:10.3390/genes8120365)
Supplement: Supplementary file 1 [file genes-08-00365-s001.zip › Supplementary File(s)/Table S1.docx]

**Table S1**:List of the SABATH genes from other species

| **No** | **Species** | ***Name*** | **GenBank accession** |
| --- | --- | --- | --- |
| 1 | *Oryza sativa* | *OsBSMT1* | XM467504 |
| 2 | *Oryza sativa* | *OsBISAMT* | AY524975 |
| 3 | *Oryza sativa* | *OsIAMT* | EU375746 |
| 4 | *Coffea arabica* | *CaCCS1* | AB086414 |
| 5 | *Coffea arabica* | *CaXMT1* | AB048793 |
| 6 | *Coffea arabica* | *CaDXMT1* | AB084125 |
| 7 | *Cucumis sativus* | *CsSAMT* | BAB84353 |
| 8 | *Antirrhinum majus* | *AmBAMT* | AF198492 |
| 9 | *Antirrhinum majus* | *AmSAMT* | AF515284 |
| 10 | *Clarkia breweri* | *CbSAMT* | AF133053 |
| 11 | *Coffea canephora* | *CcDXMT* | DQ422955 |
| 12 | *Coffea canephora* | *CcXMT* | DQ422954 |
| 13 | *Nicotiana alata* | *NaSAMT* | GU014482 |
| 14 | *Nicotiana gossei* | *NgNAMT* | GU169286 |
| 15 | *Nicotiana suaveolens* | *NsSAMT* | GU014479 |
| 16 | *Nicotiana suaveolens* | *NsBSMT* | AJ628349 |
| 17 | *Petunia×hybrida* | *PhBSMT1* | AY233465 |
| 18 | *Petunia×hybrida* | *PhBSMT2* | AY233466 |
| 19 | *Populus trichocarpa* | *PtIAMT* | XM_002298807 |
| 20 | *Stephanotis floribunda* | *SfSAMT* | AJ308570 |
| 21 | *Solanum lycopersicum* | *SlSAMT* | GU299532 |
| 22 | *Zea mays* | *ZmAAMT1* | HM242245 |
| 23 | *Zea mays* | *ZmAAMT2* | HM242244 |
| 24 | *Atropa belladonna* | *AbSAMT* | AB049752 |
