# Supplementary material for: Genome-Wide Comprehensive Analysis the Molecular Phylogenetic Evaluation and Tissue-Specific Expression of SABATH Gene Family in Salvia miltiorrhiza
Source: Genes (Basel). 2017 Dec 5;8(12):365. doi: 10.3390/genes8120365 (PMC5748683; doi:10.3390/genes8120365)
Supplement: Supplementary file 1 [file genes-08-00365-s001.zip › Supplementary File(s)/Table S2.docx]

**Table S2:** Primers for qRT-PCR

| **Gene** | **Primer name** | **Sequence(5'-3')** |
| --- | --- | --- |
| *SMil_00001154* | SMil_00001154-S | GGCGGGTGGGATGATCGTAA |
|  | SMil_00001154-A | CATGTCCGCAAGGAGGGACT |
| *SMil_00001155* | SMil_00001155-S | TCCTCGCTTCACTGGCTGTC |
|  | SMil_00001155-A | CGAACTGATCCGCGTAAGCC |
| *SMil_00003309* | SMil_00003309-S | GACGCCGACTCTATGGCTGT |
|  | SMil_00003309-A | GGTCGATGGAGGAGGAAGGC |
| *SMil_00003310* | SMil_00003310-S | TCGGATCGGCAACGACTTCA |
|  | SMil_00003310-A | CCAGTGCATCGCAACAGAGG |
| *SMil_00007297* | SMil_00007297-S | GCTCAACGCCGGGAAAGATG |
|  | SMil_00007297-A | GGAGAGCCAGTGCAGACCAT |
| *SMil_00007747* | SMil_00007747-S | GTTGTGCATGGCGGAGTTGG |
|  | SMil_00007747-A | TTCCGGCTCGACTCGTACAC |
| *SMil_00007772* | SMil_00007772-S | ACGGCCACCCGTCTTACTTC |
|  | SMil_00007772-A | TTGTCCACCACCTCCTTCGG |
| *SMil_00008156* | SMil_00008156-S | GCAGAATCACACCGGGCATC |
|  | SMil_00008156-A | TGCAGGCACCACCAGAATGA |
| *SMil_00008666* | SMil_00008666-S | GACGTGGAATGCCGGTAGGA |
|  | SMil_00008666-A | ATGATCCCACCCGCCACAAT |
| *SMil_00010605* | SMil_00010605-S | GCACAAATCAGCCGCCTCTT |
|  | SMil_00010605-A | TGGGACCGTAGAGAGCCAGT |
| *SMil_00015152* | SMil_00015152-S | GCGGTGGCGGGTTCTTTCTA |
|  | SMil_00015152-A | TTCCACGCCGGAGATCCTTC |
| *SMil_00016117* | SMil_00016117-S | AATTTGCCCGGCACAAGTGG |
|  | SMil_00016117-A | CCCGTCATCAGCACCACCAT |
| *SMil_00017556* | SMil_00017556-S | GTCGTTGGCGGACTTCAACC |
|  | SMil_00017556-A | CCGTAGAAGGAACCCGGCAT |
| *SMil_00018848* | SMil_00018848-S | GACGCGTATCGGGCTCAGTA |
|  | SMil_00018848-A | AGCGTGACCGATGCTAGAGT |
| *SMil_00020191* | SMil_00020191-S | AGATTCGGCGTGGTCGACTT |
|  | SMil_00020191-A | AGGGAACAATCTGCCGTGGA |
| *SMil_00020192* | SMil_00020192-S | GCCATGGAAGGAACTCTGGCA |
|  | SMil_00020192-A | TGCGCTTGAGCAGAGCAAAG |
| *SMil_00020193* | SMil_00020193-S | CTATTCCTCGGCCGCACTCA |
|  | SMil_00020193-A | GGGCGCCGGAGTAGTGAAT |
| *SMil_00021640* | SMil_00021640-S | AGGTGGCAAAGGCGAAGCTA |
|  | SMil_00021640-A | GCCGTCCAGGGTTTCTCTCA |
| *SMil_00021702* | SMil_00021702-S | TCCCGCGTAACTCCATCACC |
|  | SMil_00021702-A | GTGAATTCTGCCCTCGTTCGG |
| *SMil_00021703* | SMil_00021703-S | AACAAGCTGTTGGCCTCCCT |
|  | SMil_00021703-A | GAATGCGCCAAAGTGATGCG |
| *SMil_00022020* | SMil_00022020-S | GCGTGTTGTGGAGGATGCAC |
|  | SMil_00022020-A | TGAAGTCGTTGCCGATCCGA |
| *SMil_00022021* | SMil_00022021-S | TGCATTGGCTGTCCAAGTTGC |
|  | SMil_00022021-A | GGAAGCGCCCATGTAGTGGA |
| *SMil_00022342* | SMil_00022342-S | CGGTGGTCAAAGCCTATGCG |
|  | SMil_00022342-A | CCGGGCACCACGATTACGAT |
| *SMil_00022343* | SMil_00022343-S | CCATCTCGGCGTCTCTCTCC |
|  | SMil_00022343-A | GCTGTTACCGCGGTTGTTGT |
| *SMil_00023670* | SMil_00023670-S | TCCCACAAGAACTGAAGGCGAT |
|  | SMil_00023670-A | CTGCCCTGAGGTATGTGGCT |
| *SMil_00025720* | SMil_00025720-S | GTCTGACGCGGTGGTCAAAG |
|  | SMil_00025720-A | CCGGGCACCACCATCACTAT |
| *SMil_00026995* | SMil_00026995-S | GGCCTCGGAGATCAGATGGG |
|  | SMil_00026995-A | ACTTCTCGAACAGCCGCTCA |
| *SMil_00028867* | SMil_00028867-S | GCGCTTGATGCAGCTACTGT |
|  | SMil_00028867-A | TGGAGAGCCTGAAAGCAGTGT |
| *SMil_00028890* | SMil_00028890-S | TCAGCTTGCTCCCACCTGAC |
|  | SMil_00028890-A | CCTCCGTTCCAAGCCCAAGA |
| *SMil_00030124* | SMil_00030124-S | GCCACAATAGGGGAGGTGAG |
|  | SMil_00030124-A | AAAGTGCGCGCTGAAAACTC |
| *Sm**β-actin* | Smβ-actin-S | AGGAACCACCGATCCAGACA |
|  | Smβ-actin-A | GGTGCCCTGAGGTCCTGTT |
