# Supplementary material for: Genome-Wide Comprehensive Analysis the Molecular Phylogenetic Evaluation and Tissue-Specific Expression of SABATH Gene Family in Salvia miltiorrhiza
Source: Genes (Basel). 2017 Dec 5;8(12):365. doi: 10.3390/genes8120365 (PMC5748683; doi:10.3390/genes8120365)
Supplement: Supplementary file 1 [file genes-08-00365-s001.zip › Supplementary File(s)/Table S3 .docx]

**Table S3:** Gene features of *SmSABATH*

| Gene ID | Gene length(bp) | cDNA length(bp) | Protein(aa) | Mw(Da) | p*I* |
| --- | --- | --- | --- | --- | --- |
| *SMil_00001154* | 1239 | 1029 | 342 | 37773.2 | 5.64 |
| *SMil_00001155* | 2577 | 1092 | 363 | 40049.8 | 5.86 |
| *SMil_00003309* | 3463 | 849 | 282 | 29640.4 | 6.30 |
| *SMil_00003310* | 1482 | 1041 | 346 | 38375.0 | 5.20 |
| *SMil_00007297* | 1914 | 1149 | 382 | 43163.3 | 5.20 |
| *SMil_00007747* | 1123 | 1053 | 350 | 39393.1 | 5.41 |
| *SMil_00007772* | 4392 | 1125 | 374 | 40944.2 | 5.64 |
| *SMil_00008156* | 1038 | 795 | 264 | 29111.6 | 9.58 |
| *SMil_00008666* | 1899 | 765 | 254 | 28149.0 | 5.26 |
| *SMil_00010605* | 2585 | 1080 | 359 | 40910.7 | 6.84 |
| *SMil_00015152* | 1943 | 687 | 228 | 25125.5 | 5.64 |
| *SMil_00016117* | 2694 | 984 | 327 | 36416.3 | 5.68 |
| *SMil_00017556* | 3731 | 1170 | 389 | 43519.9 | 5.98 |
| *SMil_00018848* | 1426 | 1035 | 344 | 37857.1 | 5.80 |
| *SMil_00020191* | 2072 | 909 | 302 | 32979.1 | 6.11 |
| *SMil_00020192* | 5075 | 1020 | 339 | 37268.9 | 5.06 |
| *SMil_00020193* | 1932 | 1035 | 344 | 37956.4 | 5.22 |
| *SMil_00021640* | 1836 | 1185 | 394 | 43299. 0 | 5.50 |
| *SMil_00021702* | 1560 | 846 | 281 | 30889.8 | 5.03 |
| *SMil_00021703* | 1045 | 975 | 324 | 35922.2 | 6.08 |
| *SMil_00022020* | 1010 | 561 | 186 | 20007.7 | 5.68 |
| *SMil_00022021* | 1234 | 993 | 330 | 36305.3 | 5.39 |
| *SMil_00022342* | 5649 | 1134 | 377 | 40465.8 | 6.37 |
| *SMil_00022343* | 1786 | 1068 | 355 | 39553.3 | 5.34 |
| *SMil_00023670* | 2205 | 1293 | 430 | 47698.3 | 7.15 |
| *SMil_00025720* | 1293 | 588 | 195 | 21928.0 | 6.49 |
| *SMil_00026995* | 2333 | 1089 | 362 | 40481.2 | 5.71 |
| *SMil_00028867* | 1538 | 915 | 304 | 33913.5 | 5.72 |
| *SMil_00028890* | 1531 | 1044 | 347 | 39029.5 | 5.51 |
| *SMil_00030124* | 1460 | 1020 | 339 | 37170.2 | 6.02 |
