# Supplementary material for: Genome-Wide Comprehensive Analysis the Molecular Phylogenetic Evaluation and Tissue-Specific Expression of SABATH Gene Family in Salvia miltiorrhiza
Source: Genes (Basel). 2017 Dec 5;8(12):365. doi: 10.3390/genes8120365 (PMC5748683; doi:10.3390/genes8120365)
Supplement: Supplementary file 1 [file genes-08-00365-s001.zip › Supplementary File(s)/Table S4.docx]

**Table S4:** Normal expression sequences of 13 motifs identified in 30 *SmSABATH* proteins

| **Motif** | **Length(aa)** | **Frequency** | **Normal expression sequences** |
| --- | --- | --- | --- |
| 1 | 28 | 28 | GRLFPS[SN]S[ILV]H[IFV]A[YH][SC]SF[AS]LHWLSK[VL]PE[GE][LV] |
| 2 | 29 | 28 | [KR]AYADQFE[ER]D[ML][EG]IFL[RS][AC]RA[EQ]EIVAGG[LM][MI]V |
| 3 | 25 | 27 | SXK[LP]EFQVF[FL]ND[LQ][IP][GS]NDFNTLF[AQ]SL |
| 4 | 29 | 25 | T[RT]FA[VI]ADLGCS[VS]GPNT[FL]YA[MV][EQ]S[IL]IE[AT]V[QE]Q |
| 5 | 50 | 24 | L[GE]S[SAI]L[MI]D[ML][VA]KEGV[IVL][EA][EQK]EK[VI]DSFN[ILV]PIY[FAY]P[SCT]IE[ED][VLM]RRV[VI]EK[NE]G[SC]FEI[EV][KR]ME |
| 6 | 50 | 12 | [MA][EA][ES][SA]CPMNGGDGTYSY[SA]KNST[LWG]QR[DS][GV]A[SR]AV[KE]D[AL]INEA[LV]M[EK]NL[DE][IL][ES]KLLCG |
| 7 | 21 | 14 | K[DG]SPAWNAGRIHY[ST]GA[SP]DAVV |
| 8 | 15 | 28 | [QP]GRNYFAA[AG]V[PA]GSF[HY] |
| 9 | 31 | 19 | DA[EA]NV[VAI][MK]H[LM]RAVME[GP][MT][LM][AT]NHFG[ES][ESG][IV][VM][ED][QK][LV]F[EA] |
| 10 | 28 | 12 | M[KN]GGDG[EA]XSY[AS]KNSQ[YL]Q[KR]Q[AV][SA][DS][AS][MV]K[DEH][LIM]I |
| 11 | 14 | 14 | [ILM][LVI][MV]P[GA][VT]P[DN][GR][VD][VS][QDH][HY][DE] |
| 12 | 19 | 7 | RAMQ[LQR]KLHF[AT][HQR][MIL]L[DG]S[AMT]G[DFI][HK] |
| 13 | 15 | 10 | N[EKA][GK][RKS]I[HY][YIG][SNT]G[AR][SGP][EPD][ADE]VV |
