# Supplementary material for: Genome-Wide Comprehensive Analysis the Molecular Phylogenetic Evaluation and Tissue-Specific Expression of SABATH Gene Family in Salvia miltiorrhiza
Source: Genes (Basel). 2017 Dec 5;8(12):365. doi: 10.3390/genes8120365 (PMC5748683; doi:10.3390/genes8120365)
Supplement: Supplementary file 1 [file genes-08-00365-s001.zip › Supplementary File(s)/Table S6 .docx]

**Table S6:** Ka/Ks and divergence analysis of SABATH paralogous in *S.miltiorrhiza*

| **Genes** | **Ka** | **Ks** | **Ka/Ks** | **Purifying selection** | **Subgroup** | **Protein identity (%)** |
| --- | --- | --- | --- | --- | --- | --- |
| *SMil_00003309*  *SMil_00022342* | 0.0213 | 0.0561 | 0.3798 | yes | GroupA | 70.79 |
| *SMil_00020191*  *SMil_00020192* | 0.1188 | 0.2174 | 0.5465 | yes | GroupA | 72.06 |
| *SMil_00003310*  *SMil_00022020* | 0.0594 | 0.1047 | 0.5674 | yes | GroupA | 47.11 |
| *SMil_00008666*  *SMil_00025720* | 0.1091 | 0.1414 | 0.7715 | yes | GroupA | 59.02 |
| *SMil_00001154*  *SMil_00022343* | 0.0612 | 0.1539 | 0.3975 | yes | GroupA | 82.91 |
| *SMil_00007297*  *SMil_00010605* | 0.2465 | 2.2910 | 0.1076 | yes | GroupB | 59.48 |
| *SMil_00007747*  *SMil_00026995* | 0.2202 | 1.4046 | 0.1567 | yes | GroupB | 61.79 |
| *SMil_00007772*  *SMil_00021640* | 0.1419 | 1.1464 | 0.1238 | yes | GroupB | 70.60 |
| *SMil_00023670*  *SMil_00028890* | 0.3835 | 1.5018 | 0.2553 | yes | GroupB | 40.47 |
| *SMil_00008156*  *SMil_00021702* | 0.4017 | 0.7231 | 0.5556 | yes | GroupC | 32.84 |
| *SMil_00028867*  *SMil_00030124* | 0.1220 | 0.3008 | 0.4055 | yes | GroupC | 69.62 |
