# Supplementary material for: Genome-Wide Comprehensive Analysis the Molecular Phylogenetic Evaluation and Tissue-Specific Expression of SABATH Gene Family in Salvia miltiorrhiza
Source: Genes (Basel). 2017 Dec 5;8(12):365. doi: 10.3390/genes8120365 (PMC5748683; doi:10.3390/genes8120365)
Supplement: Supplementary file 1 [file genes-08-00365-s001.zip › Supplementary File(s)/Table S7 .docx]

**Table S7:** The coefficient of Type-I functional divergence (*θ*_I_) from pairwise comparisons between *SmSABATH* groups

| **Category** | **Coefficient of typeⅠfunctional divergence (*θ*_I_)±standard error** | **LRT(likelihood ratio statistic )** | **P-value** | **Positive selection sites (0.8 > *Qk* > 0.67)** | **Positive selection sites (*Qk* > 0.8)** |
| --- | --- | --- | --- | --- | --- |
| Group A vs. Group B | 0.683167±0.321242 | 4.851335 | 0.016727^*^ | 262、315、317 | 238、263、321 |
| Group A vs. Group C | 0.286372±0.337748 | 2.861579 | 0.198275. | 310、320、 | 244 |
| Group B vs. Group C | 0.675427±0.314513 | 9.895537 | 0.015877^*^ | 310、264、242、308、316、262、238、239、243、261 | 244、263、309、313、321、320、312、315 |
