# Supplementary material for: Genome-Wide Comprehensive Analysis the Molecular Phylogenetic Evaluation and Tissue-Specific Expression of SABATH Gene Family in Salvia miltiorrhiza
Source: Genes (Basel). 2017 Dec 5;8(12):365. doi: 10.3390/genes8120365 (PMC5748683; doi:10.3390/genes8120365)
Supplement: Supplementary file 1 [file genes-08-00365-s001.zip › Supplementary File(s)/Table S8.docx]

**Table S8:** The coefficient of Type-Ⅱfunctional divergence (*θ*_Ⅱ_) from pairwise comparisons between *SmSABATH* groups

| **Category** | **Coefficient of type II functional divergence (*θ*_II_)±standard error** | **P-value** | **Positive selection sites**  **(*Qk* > 0.9)** | **Positive selection sites (*Qk* > 1.0)** |
| --- | --- | --- | --- | --- |
| Group A vs. Group B | 0.041610±0.520426 | 0.468158. | 0 | 0 |
| Group A vs. Group C | 0.117169±0.354826 | 0.370624. | 238 | 309、317、265 |
| Group B vs. Group C | -0.244453±0.500169 | 0.312775 | 0 | 0 |
