# Supplementary figures and images for: Genome-Wide Comprehensive Analysis the Molecular Phylogenetic Evaluation and Tissue-Specific Expression of SABATH Gene Family in Salvia miltiorrhiza
Source: Genes (Basel). 2017 Dec 5;8(12):365. doi: 10.3390/genes8120365 (PMC5748683; doi:10.3390/genes8120365)

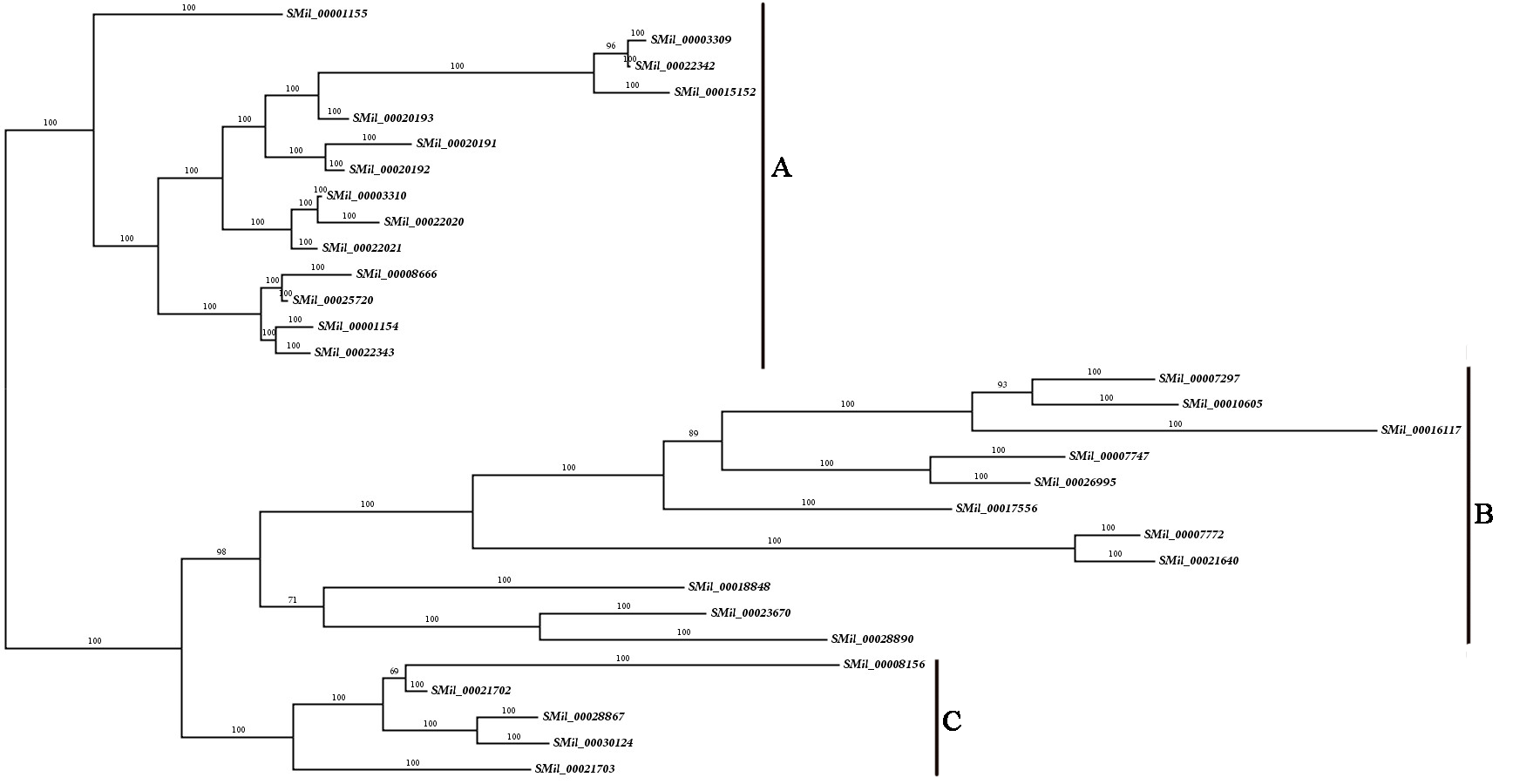

Supplement: Supplementary file 1 [file genes-08-00365-s001.zip › Supplementary File(s)/Fig S1.tif]

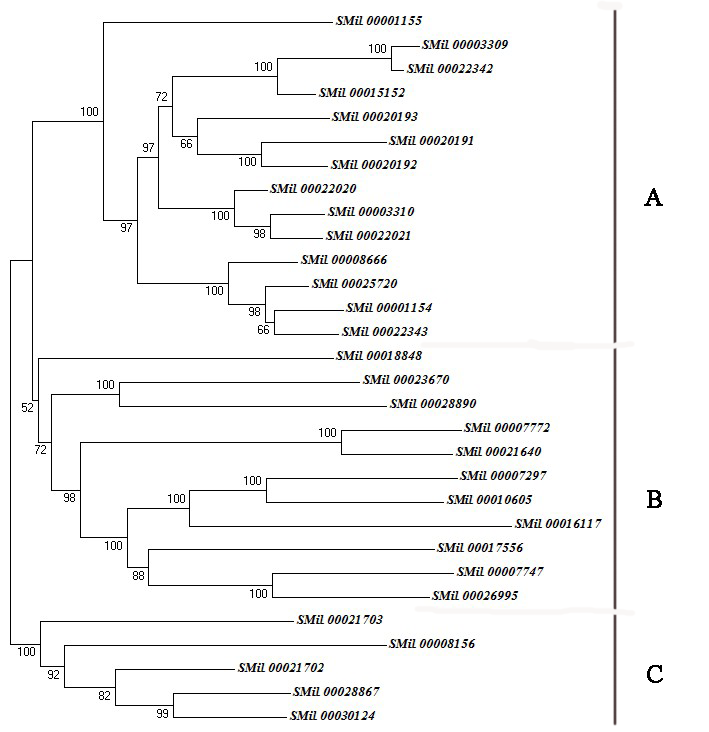

Supplement: Supplementary file 1 [file genes-08-00365-s001.zip › Supplementary File(s)/Fig S2.tif]
