# Supplementary material for: Genome-Wide Comprehensive Analysis the Molecular Phylogenetic Evaluation and Tissue-Specific Expression of SABATH Gene Family in Salvia miltiorrhiza
Source: Genes (Basel). 2017 Dec 5;8(12):365. doi: 10.3390/genes8120365 (PMC5748683; doi:10.3390/genes8120365)
Supplement: Supplementary file 1 [file genes-08-00365-s001.zip › Supplementary File(s)/Table S5 .doc]

**Table S5:** The SABATH protein motif diagram of *S.miltiorrhiza*

| Genes | E-value | Motif diagram |
| --- | --- | --- |
| *SMil_00001155* | 2.5e-223 | [6][4][3][8][1][7][2][11][5][9][12] |
| *SMil_00003309* | 1.3e-154 | [6][4][3][8][1][11] |
| *SMil_00022342* | 4.2e-218 | [6][4][3][8][1][13][2][11] |
| *SMil_00015152* | 2.7e-165 | [6][4][1][13][2][11] |
| *SMil_00020193* | 7.9e-296 | [6][4][3][8][1][7][2][11][5][9][12] |
| *SMil_00020191* | 1.2e-167 | [10][8][1][7][2][11][5][9] |
| *SMil_00020192* | 2e-283 | [6][4][3][8][1][7][2][11][5][9][12] |
| *SMil_00003310* | 0 | [6][4][3][8][1][7][2][11][5][9][12] |
| *SMil_00022020* | 6.5e-161 | [6][3][8][1][7][2] |
| *SMil_00022021* | 6e-285 | [6][4][3][8][1][7][2][5][9][12] |
| *SMil_00008666* | 1.4e-218 | [6][4][3][8][1][7][2][11] |
| *SMil_00025720* | 4.6e-146 | [3][8][1][7][2][11][12] |
| *SMil_00001154* | 1.4e-230 | [4][3][8][1][7][2][11][5][9][12] |
| *SMil_00022343* | 4.2e-218 | [6][4][3][8][1][13][2][11] |
| *SMil_00007297* | 1e-126 | [10][4][3][8][1][13][2][5][9] |
| *SMil_00010605* | 4.1e-129 | [10][4][3][8][1][13][2][5][9] |
| *SMil_00016117* | 4.8e-70 | [4][3][8][2][5][9] |
| *SMil_00007747* | 2.4e-121 | [4][3][8][1][13][2][5][9] |
| *SMil_00026995* | 1.5e-143 | [10][4][3][8][1][13][2][5][9] |
| *SMil_00017556* | 2.8e-147 | [10][4][3][8][1][13][2][5][9] |
| *SMil_00007772* | 1.9e-131 | [10][4][3][8][1][7][2][5][9] |
| *SMil_00021640* | 2.9e-136 | [10][4][3][8][1][7][2][5][9] |
| *SMil_00018848* | 5.1e-160 | [10][4][3][8][1][13][2][5][9] |
| *SMil_00023670* | 5.6e-178 | [6][4][3][8][1][7][2][5][9] |
| *SMil_00028890* | 3.4e-138 | [10][4][3][8][1][7][2][5][9] |
| *SMil_00008156* | 9.8e-70 | [2][11][5][9] |
| *SMil_00021702* | 8.5e-196 | [6][3][8][1][13][2][11][5] |
| *SMil_00028867* | 6e-167 | [10][3][8][1][13][2][11][5][9] |
| *SMil_00030124* | 2.4e-201 | [10][4][3][8][1][13][2][11][5][9] |
| *SMil_00021703* | 4.2e-136 | [4][3][8][1][13][2][11][5][9] |

**A**

**C**

**B**
